# Intuitive Thinking is Associated with Stronger Belief in Physiognomy and Confidence in the Accuracy of Facial Impressions

**DOI:** 10.1007/s10919-025-00497-w

**Published:** 2025-10-04

**Authors:** Bastian Jaeger, Anthony M. Evans, Mariëlle Stel, Ilja van Beest

**Affiliations:** 1 Tilburg University, Tilburg, Netherlands; 2 University of Twente, Enschede, Netherlands

**Keywords:** Physiognomy, Intuition, Thinking style, Lay beliefs, Trait inferences

## Abstract

Physiognomy, the idea that a person’s character is reflected in their facial features, has a long history in scholarly thought. Although now widely regarded as pseudoscience in academic circles, recent work suggests that laypeople hold physiognomic beliefs and that belief endorsement is associated with support for facial profiling technology and other outcomes. Here, we build on previous work and investigate *who* believes in physiognomy. In four studies (three preregistered), we (1) assess the prevalence of physiognomic beliefs across different sociodemographic groups, and (2) investigate its psychological correlates. In a large, representative sample of the Dutch population (Study 1, n = 2624), about 50% of participants at least somewhat endorsed physiognomic beliefs. Endorsement of physiognomic beliefs varied little as a function of gender, age, education, and income. Across different measures of thinking styles and other lay beliefs, we found that physiognomic beliefs were most strongly related to how much people trust their intuitions—an association that emerged consistently with British (Study 2, n = 224), Nigerian (Study 3, n = 147), and Dutch participants (Study 4, n = 388). Participants who scored higher on faith in intuition were also more confident in the accuracy of their face-based trustworthiness impressions. In sum, the present studies suggest that lay beliefs in physiognomy are (a) common, (b) similarly endorsed across various socio-demographic groups, and (c) associated with an intuitive thinking style.

**Supplementary Information:**

The online version contains supplementary material available at 10.1007/s10919-025-00497-w.

## Introduction

People spontaneously judge the character of strangers based on their facial appearance (Todorov et al., 2015). Although facial impressions of trustworthiness, extraversion, intelligence, and many other traits that were studied in previous research show little to no accuracy (Bonnefon et al., 2017; Jaeger et al., 2024; Mitchem et al., 2015), people are still relatively confident in their impressions (Ames et al., 2010; Hassin and Trope, 2000; Jaeger et al., 2024) and rely on them when making consequential decisions (Olivola et al., 2014). For example, facial impressions influence voting decisions (Olivola and Todorov, 2010), personnel selection (Gomulya et al., 2017), and criminal sentencing (Zebrowitz and McDonald, 1991). People even rely on facial impressions when they have access to more diagnostic information (Jaeger et al., 2019; Rezlescu et al., 2012) and when they are explicitly instructed to discount a person’s appearance (Jaeger et al., 2020). To explain why people rely on their first impressions so persistently, recent research has explored the role of lay beliefs in physiognomy, the idea that a person’s character is reflected in their facial appearance (Jaeger et al., 2020). The present studies build on previous work on this topic (e.g., Madan et al., 2022; Suzuki et al., 2017, 2022) and examine the prevalence and psychological correlates of belief in physiognomy.

### Lay Beliefs in Physiognomy

The theory and practice of physiognomy can be traced back to both Ancient Greece (Aristotle, 1936) and Ancient China (McCarthy, 2007). Early writings proposed that the size and orientation of facial features reflect their frequent use (e.g., a disagreeable person who frowns a lot will have lowered eyebrows), or that resemblances between humans and other animals point to shared psychological attributes (Aristotle, 1936). These ideas achieved mainstream appeal in Europe after Lavater published his extensive *Essays on Physiognomy* at the end of the 1700s (Lavater, 1775). Guidebooks on physiognomy were popular; various illustrations allowed people to match different features of a person’s appearance with the character traits that they were supposedly revealing (Alley, 1988).

Toward the end of the 1800s, applications of physiognomy, and the related practice of phrenology in which the size and shape of a person’s skull is used to judge their character and abilities, took a more sinister turn. These practices were used in an attempt to identify “deficient” or undesired members of society (Alley, 1988). For example, British teachers were encouraged to identify “feeble-minded” children who would not benefit from education by their appearance, including prompts like “What is the appearance of the child—stupid or bright?” (Report of the Departmental Committee on Defective and Epileptic Children, 1898). Lombroso’s study of criminals aimed to demonstrate the possibility of identifying deviant individuals by their facial appearance (Lombroso, 1911). Physiognomic measurement was also an important tool of the eugenics movement, where racial differences in appearance were systematically documented in an attempt to demonstrate the inferiority of certain groups (e.g., Galton, 1891). Similar ideas can be found in many racially motivated ideologies, including in Nazi Germany, where physiognomic differences of Black or Jewish individuals were highlighted to justify their supposed inferiority (Gray, 2004).

Since the publication of Lavater’s work, various scholars had voiced their skepticism of physiognomy in the academic literature, but the idea remained relatively popular well into the 1900s, when more systematic and rigorous psychological studies showed that many claimed links between facial features and personality traits were not supported by the empirical evidence (Alley, 1988). Although physiognomy is now widely viewed as pseudoscience in academic circles (Todorov, 2017), recent research has started to explore the antecedents and consequences of belief in physiognomy among laypeople. Lay beliefs in physiognomy (also referred to as the *appearance reveals character* lay theory; Madan et al., 2022) capture an individual’s belief that a person’s character traits are reflected in their appearance, most commonly their facial appearance (Suzuki et al., 2017). This belief has been measured with general items (e.g., “People’s appearance is a mirror of their character”; Madan et al., 2022) or trait-specific items (e.g., “I know an immoral person when I see their face”; Suzuki et al., 2017).

Prior work suggests that people who more strongly believe in physiognomy also make more extreme character judgments based on a person’s facial appearance (Suzuki et al., 2017, 2022) and that they are more confident in the accuracy of their impressions (Jaeger et al., 2022; Madan et al., 2022). Going beyond impression formation, lay beliefs in physiognomy are also associated with how people use (or condone the use) of facial appearance in judgment and decision-making. Madan and colleagues (2022) found that stronger beliefs in physiognomy were related to stronger support for the use of facial profiling algorithms in hiring, policing, and other contexts. In a similar vein, people who more strongly endorsed physiognomic beliefs rated personal photos on CVs as more useful for making hiring decisions, and reliance on photos as more appropriate and effective (Jaeger et al., 2022). Another study found that belief in physiognomy was positively related to how much participants relied on their trustworthiness impressions in a simulated criminal sentencing task (Jaeger et al., 2020). Thus, lay beliefs in physiognomy may be one explanation for the widespread confidence in (and reliance on) first impressions, despite their low accuracy.

### Who Believes in Physiognomy?

Prior studies have tested associations between physiognomic beliefs and various other lay beliefs and individual differences. One general pattern that has emerged from these studies is an association between belief in physiognomy and a suite of lay belief in the idea that character traits are a fundamental, inherent, and relatively unchangeable aspects of a person’s identity. In studies with participants from the United States and Japan (Suzuki et al., 2017), belief in physiognomy was positively related to the belief that personality traits are biologically determined. A similar association was observed for so-called entity beliefs (also referred to as a “fixed” mindset) about personality —beliefs that personality traits are fixed and immutable rather than malleable (Madan et al., 2022; Suzuki et al., 2017).^1^

We propose that belief in physiognomy may also be related to individual differences in thinking styles. Thinking styles (sometimes referred to as epistemic motivation or cognitive styles) capture individual differences in general cognitive processes, such as how much people trust their intuitions, how much they enjoy thinking, and the extent to which they keep an open mind for evidence that contradict their beliefs (Epstein et al., 1996; Pennycook et al., 2015; Stanovich and Toplak, 2023). Trait impressions from faces are formed quickly and effortlessly (Stewart et al., 2012; Willis and Todorov, 2006) and this accessibility may make them intuitively appealing. We therefore test whether the endorsement of physiognomic beliefs is more prevalent among people who tend to trust their intuitions (Epstein et al., 1996). That is, the quick and intuitive nature by which trait impressions arise could lead people who generally attach more weight to their intuitions to feel more confident in their specific impressions, and, more generally, in the idea that character is reflected in faces.

Relatedly, some people are more prone to override their intuitive responses with more analytic and reflective responses, which is captured by tasks such as the cognitive reflection test (Frederick, 2005; Pennycook et al., 2015). Given the quick accessibility of first impressions when perceiving others, the idea that these impressions arise because they are a valid guide to a person’s character may be intuitively appealing. It is plausible that many people would reject this idea when reflecting on it (when prompted, lay people often find it difficult to articulate why the facial features that make someone look trustworthy or intelligent should be associated with the person’s actual trustworthiness or intelligence). However, this process might not occur for people who are less likely to reflect on their reasons for endorsing physiognomic beliefs, leading the belief to persist. We therefore tested whether physiognomic beliefs are negatively related to cognitive reflection.

Finally, people vary in their need to form evaluative judgments (Jarvis and Petty, 1996). If people form personality impressions based on others’ facial appearance (which is usually an easily available cue) to satisfy this tendency, endorsement of physiognomic beliefs may justify this behavior. We therefore tested whether physiognomic beliefs are correlated with individual differences in the need to evaluate.

### The Present Studies

Initial evidence for a relation between thinking styles and lay beliefs in physiognomy was provided by a study with U.S. American participants, which found that beliefs in physiognomy were correlated with faith in intuition, but not with cognitive reflection (Suzuki et al., 2022). The primary goal of the current studies is to extend this work. Across four studies (three preregistered), we examine the role of thinking styles, other lay beliefs, and socio-demographic variables in explaining variation in the endorsement of physiognomic beliefs. In Study 1, we first investigate whether we could replicate previous findings showing that physiognomic beliefs are correlated with entity beliefs about personality and beliefs in biological determinism in a large, representative sample of the Dutch population (n = 2624). In Study 2 (n = 224), we extend our analyses to the role of thinking styles. We examine associations between physiognomic beliefs and measures of faith in intuition, cognitive reflection, and the need to evaluate (next to measures of different lay beliefs) in a sample of British participants. Study 3 (n = 147) and Study 4 (n = 388) test whether our main findings of Study 2 replicate with Nigerian and Dutch participants.

We extend previous work on the relation between thinking styles and belief in physiognomy in four ways. First, we test the generalizability of the association across three countries. Second, the faith in intuition scale (Epstein et al., 1996), which was used in the only previous study examining the relation between thinking styles and physiognomic beliefs (Suzuki et al., 2022), contains two items that directly reference beliefs that character can be judged from appearance (“I believe I can judge character pretty well from a person's appearance”, “My initial impressions of people are almost always right”). It is not clear if the previously observed association with physiognomic beliefs is driven by these items. In our studies, we test if positive associations still emerge when these two items are omitted. Third, it is plausible that different measures of thinking styles and lay beliefs are correlated. In Study 2, we regress physiognomic beliefs on six variables (faith in intuition, cognitive reflection, the need to evaluate, belief in the biological determinism of personality, belief in the entity theory of personality, and belief in a just world) to test which variables show a unique association with physiognomic beliefs when controlling for the others. We also test which measure emerges as the strongest predictor of physiognomic beliefs. Fourth, going beyond lay beliefs in physiognomy, Study 4 tests the relation between thinking style and confidence in the accuracy of face-based character judgments.

All data, analysis scripts, and preregistration documents are available at the Open Science Framework (https://osf.io/s9nj8/). We report all measures, how our sample sizes were determined, and all data exclusions. Studies 1, 2, and 4 were preregistered.

## Study 1

In Study 1, we tested whether we could replicate findings by Suzuki and colleagues (2017) on the relation between physiognomic beliefs and other lay beliefs on the nature of character traits. Specifically, we tested whether physiognomic beliefs are positively associated with entity beliefs about personality (Chiu et al., 1997) and beliefs in the biological determinism of character traits (Haslam et al., 2004). Extending the work by Suzuki and colleagues (2017), we also examined the prevalence of lay beliefs by measuring physiognomic beliefs in a large, representative sample of the Dutch population and we tested whether belief in physiognomy varies across different socio-demographic indicators (i.e., gender, age, education level, and income level).

### Methods

#### Participants

 We recruited a representative sample of 2807 Dutch participants via the LISS (Longitudinal Internet Studies of the Social Sciences) panel (Scherpenzeel and Das, 2010). The panel is based on a probability sample of Dutch households drawn from the population register. Panel members are representative of the Dutch population on indicators like gender, age, education, and income. For more information on the LISS panel, see lissdata.nl. Data from 183 participants (6.52%) who had missing data for at least one question was excluded from analysis, leaving a final sample of 2624 participants (*M*_age_ = 52.60, *SD*_age_ = 16.50, age range: 16–95; 52.52% female, 47.48% male).

A sensitivity analysis in G*Power (Faul et al., 2007) showed that this sample size afforded us 80% power to detect very small correlations between lay belief in physiognomy and other lay beliefs (*r* = .055 with α = 5%).

#### Materials and procedure

 We measured lay beliefs in physiognomy with the physiognomic belief scale (Jaeger et al., 2020, 2022). Participants were prompted to imagine seeing the passport photo of a stranger. They were asked to indicate how much they agreed with three statements (e.g., *I can learn something about a person’s personality just from looking at his or her face*) on a scale from 1 (*strongly disagree*) to 7 (*strongly agree*). Average scores across the three items constituted our measure of general physiognomic beliefs. The reliability of the scale (McDonald’s ω = 0.66). We randomized the order in which the three items were presented.^2^

Following previous investigations (Haslam et al., 2004; Suzuki et al., 2017), we measured belief in the biological determinism of personality traits by showing participants a list of nine personality traits—the same nine personality traits representing evaluations of sociability, morality, and competence that are used for the physiognomic belief scale (ω = 0.92). We asked them to rate how much each trait is based on biological nature (genes, brain structure, etc.) on a scale ranging from 0 (*not based on biological nature*) to 100 (*based on biological nature*).

Belief in the entity theory of personality was measured with eight items (e.g., “The kind of person someone is, is something basic about them, and it can’t be changed very much”; ω = 0.83) adapted from Levy and colleagues (1998). Participants indicated how much they agreed with each statement on a scale ranging from 1 (*strongly disagree*) to 6 (*strongly agree*).

Finally, using participants’ unique identification number provided by the panel, we obtained data on participants’ gender, age, education level, and income level. The income variable represented the *z*-standardized monthly net income, and the education variable distinguished between six levels of the Dutch education system: “Basisonderwijs” (primary school), “VMBO” (preparatory secondary vocational education), “HAVO/VBO” (senior general secondary education), “MBO” (secondary vocational education), “HBO” (university of applied sciences), and “WO” (academic university). Data from 278 participants (10.59%) who indicated an income of zero and 2 respondents (0.08%) whose reported income was 23.7 and 41.4 standard deviation above the mean were excluded from analysis, leaving a sample of 2344 participants for this analysis.

### Results

#### Other lay beliefs

 First, we examined correlations between physiognomic beliefs and other lay beliefs. We found a significant, positive correlation between belief in physiognomy and belief in biological determinism, *r*(2622) = 0.160, *p* < .001, 95% CI [0.122, 0.197]. Participants who believed that personality traits are biologically determined also believed that they are reflected in people’s facial appearance. We did not find a significant correlation with belief in the entity theory of personality, *r*(2622) = 0.025, *p* = .192, 95% CI [− 0.013, 0.064]. Thus, we did not find that participants who more strongly believe that personality traits are fixed and immutable also score higher on physiognomic belief.

#### Prevalence and socio-demographic characteristics

**Fig. 1:**
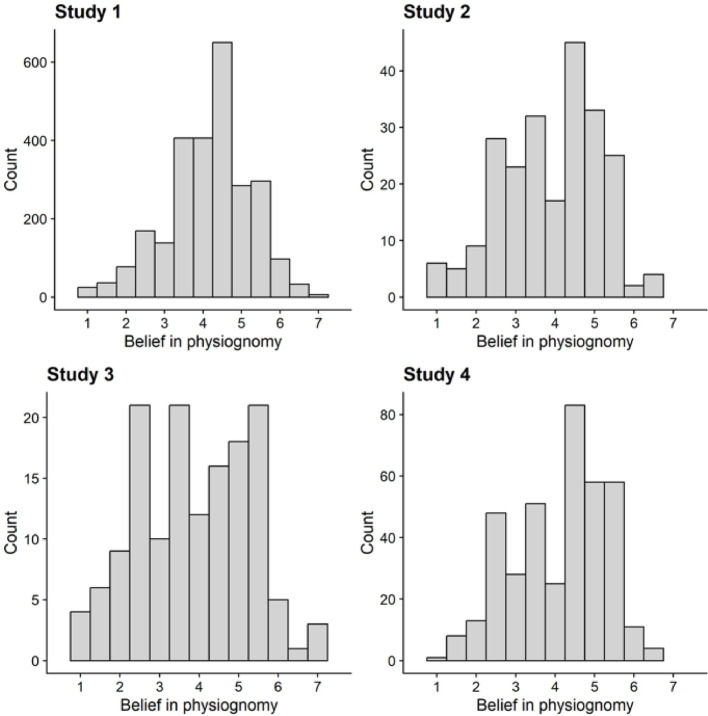
Distributions of belief in physiognomy across the studies

 Next, we examined the prevalence of physiognomic beliefs. The average score on general physiognomic belief was just above the scale midpoint (*M* = 4.17, *SD* = 1.08; see Table 1 and Fig. 1). Around half of all participants (52.10%) believed at least somewhat in physiognomy (i.e., they scored above the midpoint of the scale indicating at least a weak belief). Thus, belief in physiognomy was relatively common.

**Table 1:** Descriptive statistics for belief in physiognomy across all studies

Sample	n	ω	*M*	*SD*	% Believers
Study 1: Dutch sample (LISS panel)	2624	0.66	4.17	1.08	52.10
Study 2: British sample (Prolific)	229	0.86	3.92	1.23	47.60
Study 3: Nigerian sample (Toloka)	147	0.78	3.86	1.41	43.54
Study 4: Dutch sample (student pool)	388	0.78	4.12	1.18	55.15

Physiognomic beliefs were measured with three items that were rated on a scale from 1 (*strongly disagree*) to 7 (*strongly agree*). “% Believers” indicates the percentage of participants that scored above the midpoint of the scale (4), that is, participants who responded with slightly agree (5), agree (6), or strongly agree (7) with the propositions

We also explored relations between physiognomic beliefs and basic demographic characteristics. We regressed general physiognomic belief on gender (coded 0 for male and 1 for female), age, income (*z*-standardized net monthly income), and level of education (six levels, ranging from primary school to university degree; see Table 2, Model 1). Women scored higher on physiognomic belief than men, β = 0.104, SE = 0.048, t(2335) = 2.17, *p* = 0.030, 95% CI [0.010, 0.198], and there was a negative effect of age, β =  − 0.004, SE = 0.001, t(2335) = 3.07, *p* < .001, 95% CI [− 0.007, − 0.002]. However, these effects were very small: women’s physiognomic belief score was 0.097 standard deviations above men’s score and a ten-year age difference was associated with a 0.039 standard deviation decrease in physiognomic belief. We did not find significant associations between physiognomic beliefs and income or education.

**Table 2:** Predictors of physiognomic belief in Study 1

	Model 1			Model 2		
	β	SE	*p*	β	SE	*p*
Intercept	4.353	0.131	< 0.001	4.362	0.129	< 0.001
Female	0.104	0.048	0.030	0.126	0.047	0.008
Age	− 0.004	0.001	0.002	− 0.005	0.001	< 0.001
Net income	0.166	0.153	0.280	0.181	0.151	0.231
Highest education (2): VMBO	0.055	0.105	0.600	0.023	0.104	0.827
Highest education (3): HAVO/VBO	− 0.055	0.119	0.648	− 0.044	0.118	0.711
Highest education (4): MBO	0.053	0.105	0.615	0.040	0.104	0.703
Highest education (5): HBO	0.042	0.107	0.691	0.051	0.105	0.626
Highest education (6): WO	− 0.154	0.118	0.192	− 0.113	0.117	0.335
Entity beliefs about personality				− 0.013	0.023	0.553
Belief in bio. determinism				0.184	0.023	< 0.001

Net income, belief in biological determinism, and entity beliefs were *z*-standardized.“Basisonderwijs” (primary school) served as the reference category for highest educational attainment. “VMBO”: preparatory secondary vocational education, “HAVO/VBO”: senior general secondary education, “MBO”: secondary vocational education, “HBO”: university of applied sciences, “WO”: academic university. Categories are sorted from lower to higher educational attainment

#### Robustness check

 Finally, we tested whether the observed relation between belief in biological determinism and belief in physiognomy still emerges when controlling for socio-demographic characteristics. We regressed physiognomic beliefs on belief in biological determinism, belief in the entity theory of personality, gender, age, education level, and income level (see Table 2, Model 2). There was still a significant association between belief in biological determinism and belief in physiognomy, β = 0.184, SE = 0.023, t(2333) = 8.12, *p* < .001, 95% CI [0.140, 0.229], whereas the association between belief in the entity theory of personality and belief in physiognomy was still non-significant, β =  − 0.013, SE = 0.023, t(2333) = 0.59, *p* = .553, 95% CI [− 0.060, 0.031].

### Discussion

Replicating findings by Suzuki and colleagues (2017), we found that people who think that a person’s personality is biologically determined also scored higher on belief in physiognomy. These results suggest that belief in physiognomy may be rooted in a more general lay belief that a common factor (e.g., genetic makeup) determines both personality traits and a person’s appearance. Contrary to previous findings (Madan et al., 2022; Suzuki et al., 2017), we did not find that physiognomic beliefs were positively correlated with entity beliefs about personality, the idea that personality is an essential and fixed aspect of a person’s identity.

Our results also provided more insights into the prevalence of physiognomic beliefs. We found that belief in physiognomy was common in a large representative sample of the Dutch population, with over half of all participants at least somewhat endorsing it. Belief in physiognomy was significantly stronger among women and younger participants, but these differences were negligible in size. We found no evidence that belief endorsement varied across different levels of education or income. Thus, our results showed that physiognomic beliefs are common across different demographic groups.

## Study 2

In Study 2, we examined a broader suite of potential psychological correlates of physiognomic beliefs. We again tested whether physiognomic beliefs are positively correlated with beliefs in biological determinism and entity beliefs about personality, this time with a sample of British participants. We also examined another lay belief that has been linked to physiognomic beliefs in previous work (Suzuki et al., 2017): belief in a just world (Lipkus, 1991). This result suggests that physiognomic beliefs may also be rooted in the idea that people “get what they deserve”, with, for example, immoral people having a facial appearance that betrays their immorality to others.

The main goal of Study 2 was to investigate the correlation between thinking styles and individual differences in physiognomic beliefs. Given that trait impressions are formed spontaneously, quickly, and effortlessly (Stewart et al., 2012; Willis and Todorov, 2006) they may be an intuitively appealing input when judging others. We therefore examined associations between physiognomic beliefs and individual differences in faith in intuition (Epstein et al., 1996), cognitive reflection (Frederick, 2005; Pennycook et al., 2015), and the need to evaluate (Jarvis and Petty, 1996).

Finally, we also explored the temporal stability of belief in physiognomy. Participants completed the physiognomic belief scale at two time points with a four-week delay.

### Methods

#### Participants

 In the study of Suzuki and colleagues (2017), correlations ranging from r = 0.185 to r = 0.445 were reported. We therefore aimed to recruit 227 participants, which affords 80% power to detect a correlation of r = 0.185 (with α = 5%). We recruited 310 British Prolific workers to complete the study in exchange for £1.25. In line with our preregistration, data from 79 participants (25.48%) who failed an attention check question at the end of the study and from 2 participants (0.87%) who indicated poor or basic English proficiency were excluded from analysis, leaving a final sample of 229 participants (*M*_age_ = 35.62, *SD*_age_ = 11.86; 60.26% female, 39.30% male, 0.44% other).

To measure the temporal stability of physiognomic beliefs, we re-contacted participants after four weeks. An a priori power analysis showed that a sample size of 84 participants is required to detect a medium-sized correlation (r = 0.3) between physiognomic belief scores at both time points with 80% power (and α = 5%). Following Suzuki and colleagues (2017), we aimed to recruit a minimum of 120 participants. All 229 participants who completed wave 1 of the study were invited to participate. A total of 200 participants completed the study in exchange for £0.50. In line with our preregistration, data from 55 participants (27.50%) who failed an attention check question at the end of the study and from 16 participants (11.03%) whose responses could not be matched with data from part one were excluded from analysis, leaving a final sample of 129 participants (*M*_age_ = 38.26, *SD*_age_ = 12.16; 60.47% female, 38.76% male, 0.78% other).

#### Materials and procedure

 Belief in physiognomy (ω = 0.86), belief in the entity theory of personality (ω = 0.95), and belief in the biological determinism of personality traits (ω = 0.93) were measured as described in Study 1.

Belief in a just world was measured with seven items (e.g., “I feel that people get what they deserve”; ω = 0.91) adapted from Lipkus (1991). Participants indicated how much they agreed with each statement on a scale ranging from 1 (*strongly disagree*) to 6 (*strongly agree*).

Faith in intuition was measured with twelve items (e.g., “I trust my initial feelings about people”; ω = 0.90) adapted from Epstein and colleagues (1996). Participants indicated how much they agreed with each statement on a scale ranging from 1 (*completely false*) to 5 (*completely true*).

Cognitive reflection was measured with the seven-item cognitive reflection test (CRT; e.g., “If you are in a race and you pass the person in second place, what place are you in?”; ω = 0.76) adapted from Thomson and Oppenheimer (2016). The CRT measures the tendency to override an intuitive, but incorrect answer with a more reflective and correct one. Participants indicated their responses in a free form text box. The test was scored by adding up the number of items that were answered correctly.

The need to evaluate was measured with sixteen items (e.g., “I form opinions about everything”; ω = 0.88) adapted from Jarvis and Petty (1996). Participants indicated to what extent each item was characteristic of them on a scale ranging from 1 (*extremely uncharacteristic*) to 5 (*extremely characteristic*).

Participants completed the six measures, and the items within each measure, in a random order. We randomized whether participants completed the physiognomic belief scale before or after the other measures. For the second part of the study, which was conducted four weeks later, participants completed the physiognomic belief scale a second time.

### Results

At time 1, the average score on general physiognomic belief was just below the midpoint of our scale (*M* = 3.92, *SD* = 1.23; see Table 1 and Fig. 1). Around half of all participants (47.60%) believed at least somewhat in physiognomy (i.e., they scored above the midpoint of the scale).

#### Psychological correlates 

First, we examined the relation between physiognomic beliefs and other lay beliefs. Participants who scored higher on physiognomic beliefs also scored higher on belief in the biological determinism of personality traits, r(227) = 0.172, *p* = 0.009, 95% CI [0.026, 0.280], and belief in a just world, *r*(227) = 0.154, *p* = 0.020, 95% CI [0.025, 0.278]. As in Study 1, we did not find that physiognomic beliefs were related to belief in the entity theory of personality, *r*(227) = 0.093, *p* = .161, 95% CI [− 0.037, 0.220].

Next, we examined how physiognomic beliefs relate to different measures of thinking styles (see Table 3). Participants who scored higher on physiognomic beliefs also scored higher on faith in intuition, *r*(227) = 0.409, *p* < .001, 95% CI [0.295, 0.511]. Note that the faith in intuition scale includes two items that directly refer to the accuracy of appearance-based impressions (“My initial impressions of people are almost always right” and “I believe I can judge character pretty well from a person's appearance”). Physiognomic beliefs were still correlated with faith in intuition when these two items were omitted, *r*(227) = 0.345, *p* < .001, 95% CI [0.226, 0.454]. We did not find significant correlations between belief in physiognomy and individual differences in cognitive reflection, *r*(227) =  − .048, *p* = .551, 95% CI [− 0.168, 0.091], or the need to evaluate, *r*(227) = 0.125, *p* = .058, 95% CI [− 0.004, 0.251].

**Table 3:** Descriptive statistics and correlations for all psychological measures

Measure	*M*	*SD*	ω	Correlation					
				1	2	3	4	5	6
1. PB	3.92	1.23	0.86	–	–	–	–	–	–
2. BET	3.30	0.90	0.95	0.093	–	–	–	–	–
3. BBD	43.61	18.57	0.93	0.172***	0.229***	–	–	–	–
4. BJW	2.98	0.81	0.91	0.154*	0.017	0.104	–	–	–
5. FI	3.56	0.60	0.90	0.409***	0.054	0.253***	0.183**	–	–
6. CRT	3.73	1.91	0.76	− 0.048	0.019	− 0.037	0.016	− 0.177**	–
7. NE	3.48	0.60	0.88	0.125^†^	0.079	0.116^†^	− .0125^†^	0.370***	− 0.009

PB, physiognomic belief; BET, belief in the entity theory of personality; BBD, belief in the biological determinism of personality traits; BJW, belief in a just world; FI, faith in intuition; CRT, cognitive reflection; NE, need to evaluate

^†^*p* < .10, *p < .05, **p < .01, ***p < .001

Finally, we explored which of the measures is most strongly related to physiognomic beliefs. We estimated a multiple regression model in which we regressed physiognomic beliefs on all six measures, which were *z*-standardized prior to analysis (see Table 4). There was a positive effect of faith in intuition, β = 0.486, SE = 0.086, t(222) = 5.62, *p* < .001, 95% CI [0.315, 0.656], but no significant effects of entity beliefs about personality, β = 0.078, SE = 0.77, t(222) = 1.01, *p* = 0.312, 95% CI [− 0.074, 0.230], belief in biological determinism, β = 0.045, SE = 0.080, t(222) = 0.57, *p* = .573, 95% CI [− 0.112, 0.202], belief in a just world, β = 0.091, SE = 0.78, t(222) = 1.17, *p* = .244, 95% CI [− 0.063, 0.245], cognitive reflection, β = 0.031, SE = 0.076, t(222) = 0.41, *p* = .682, 95% CI [− 0.119, 0.181], or need to evaluate, β =  − 0.024, SE = 0.083, t(222) = 0.30, *p* = .764, 95% CI [− 0.188, 0.138].

**Table 4:** Predictors of physiognomic belief in Study 2

	β	SE	*p*
Intercept	3.920	0.075	< 0.001
Entity beliefs about personality	0.078	0.077	0.312
Belief in biological determinism	0.045	0.080	0.573
Belief in a just world	0.091	0.078	0.244
Faith in intuition	0.486	0.086	< 0.001
Cognitive reflection	0.031	0.076	0.682
Need to evaluate	− 0.025	0.083	0.764

#### Temporal stability of belief in physiognomy

Another goal of the present study was to estimate the test–retest reliability of belief in physiognomy. This part of the study was also preregistered. All Prolific workers who completed the first part of this study were eligible to participate in the second part, in which we administered the physiognomic belief scale again. The second wave was made available four weeks after the first wave.

The average score on general physiognomic belief when measured four weeks later was just below the midpoint of our scale (*M* = 3.99, *SD* = 1.18). Around half of all participants (48.84%) believed at least somewhat in physiognomy. We found a strong correlation between general physiognomic belief scores at both time points, r(127) = 0.644, *p* < .001, 95% CI [0.531, 0.736].^3^

### Discussion

The current study provided new insights into who believes in physiognomy. In line with previous work (Suzuki et al., 2017), we found that people scored higher on belief in physiognomy also scored higher on belief in the biologically determined nature of personality. Unlike previous studies (Madan et al., 2022; Suzuki et al., 2017), we did not observe a correlation between entity beliefs about personality (i.e., a “fixed” mindset) and physiognomic beliefs. Thus, we observed the same pattern of correlations as in Study 1.

The primary goal of this study was to examine the role of thinking styles. Results showed that participants who generally put more trust in their intuitions more strongly endorsed physiognomic beliefs. This association still emerged when omitting two items from the faith in intuition scale that directly reference one’s confidence in character judgments. This finding is in line with the idea that people endorse physiognomic beliefs because of the quick and intuitive way by which trait impressions from faces arise. People who generally trust their intuitions may not only show confidence in the accuracy of specific impressions that arise intuitively, but they may also endorse the more general idea that character is reflect in a person’s facial features. We did not find that physiognomic beliefs were related individual differences in cognitive reflection or the need to evaluate.

We also found that faith in intuition was positively related to belief in biological determinism and belief in a just world. Crucially, when predicting physiognomic beliefs with all individual differences measured here, both lay beliefs and measures of thinking styles, we found that only faith in intuition was significantly association with belief in physiognomy. This suggests that, at least when considering the various lay beliefs and thinking style measures that were considered here, variation in the endorsement of physiognomic beliefs is best explained by how much weight people generally attach to their intuitions.

## Study 3

In Study 3, we tested the cross-cultural generalizability of the main finding of Study 2. Specifically, we tested whether the positive association between faith and intuition and belief in physiognomy would replicate with Nigerian participants. We decided to recruit participants from Nigeria because Nigeria has a large English-speaking population, which allowed us to replicate Study 2 using the same materials, but it is culturally very different from the United Kingdom, from which participants from Study 2 were sampled. The multidimensional cultural distance metric developed by Muthukrishna and colleagues (2020) shows a large distance score of 0.224 between Nigeria and Great Britain. For comparison, other cultural distance scores for Great Britain are 0.031 for the Netherlands, 0.060 for the United States, and 0.208 for China.

### Methods

#### Participants

In Study 2, we found a correlation of r = 0.409 between faith in intuition and belief in physiognomy. As a conservative estimate, we assumed a correlation of r = 0.25 for the present study. A power analysis showed that 123 participants are required to detect a correlation of this size with 80% power (and α = 5%). We ultimately recruited 150 participants via the recruitment platform Toloka (https://toloka.ai/). Data from 3 participants (2%) who indicated that they do not live in Nigeria were excluded from analysis, leaving a final sample of 147 participants (*M*_age_ = 32.28, *SD*_age_ = 9.58; 25.85% female, 74.15% male, 0% other).

#### Materials and procedure

 Belief in physiognomy (ω = 0.75) and faith in intuition (ω = 0.87) were measured as in Study 2.

### Results

The average score on general physiognomic belief was just below the midpoint of our scale (*M* = 3.86, *SD* = 1.42; see Table 1 and Fig. 1). Around half of all participants (43.54%) believed at least somewhat in physiognomy (i.e., they scored above the midpoint of the scale).

Replicating the result of Study 2, we again found a positive correlation between faith in intuition and belief in physiognomy, *r*(145) = 0.351, *p* < .001, 95% CI [0.200, 0.485]. The positive correlation still emerged when excluding two items that directly refer to the accuracy of appearance-based impressions from the faith in intuition scale, *r*(145) = 0.301, *p* < .001, 95% CI [0.146, 0.441].

### Discussion

We again observed a positive correlation between an intuitive thinking style and belief in physiognomy, replicating the result from Study 2 (with British participants) in a sample of Nigerian participants.

## Study 4

In Studies 1–3, we measured participants’ explicit belief that they can learn something about a person’s character from their facial appearance. In Study 4 we extended our focus and also measured participants’ confidence in the accuracy of specific appearance-based character judgments. Dutch university students viewed a series of facial photographs and judged the trustworthiness of the depicted individuals. After each judgment, participants indicated how confident they were that their impression was accurate and, at the end of the study, they completed the physiognomic belief scale and the faith in intuition scale. This allowed us to test if the positive correlation between faith in intuition and belief in physiognomy observed in Studies 2 and 3 (with British and Nigerian participants) would replicate in a Dutch sample. Crucially, it also allowed us to test if people who score high on faith in intuition would also be more confident in the accuracy of specific face-based trustworthiness impressions.

### Methods

#### Participants

We aimed to collect a minimum sample of 193 participants, which would be sufficient to detect a small correlation (*r* = 0.2) with 80% power (with α = 5%). The final sample size was determined by how many participants took part in the study within approximately four weeks. Participants were drawn from the pool of first-year psychology students at a Dutch university. A total of 395 participants completed the study. In line with our preregistration, data from 2 participants (0.51%) who indicated only basic English proficiency and from 5 participants (1.27%) who always indicated the same trustworthiness rating on all trials were excluded from analysis, leaving a final sample of 388 participants (*M*_age_ = 20.17, *SD*_age_ = 2.26; 86.08% female, 13.14% male, 0.77% other).

#### Materials and procedure

 To measure confidence in facial impressions, we showed participants 20 facial photographs of individuals with neutral expressions (10 male, 10 female) taken from the Radboud Faces Database (Langner et al., 2010). The images were displayed in a random order. On each trial, participants were first asked to rate how trustworthy they think the person in the photo is on a scale that ranged from 1 (*not at all*) to 9 (*extremely*). After indicating their trustworthiness judgment, participants were asked to rate their confidence in the accuracy of their judgment on a scale that ranged from 1 (*not at all*) to 9 (*extremely*). We then averaged participants’ confidence ratings across the 20 trials (ω = 0.97).

Belief in physiognomy (ω = 0.78) and faith in intuition (ω = 0.82) were measured as in Studies 2 and 3.

### Results

The average score on general physiognomic belief was just below the midpoint of our scale (*M* = 4.11, *SD* = 1.18; see Table 1 and Fig. 1). Around half of all participants (55.15%) believed at least somewhat in physiognomy (i.e., they scored above the midpoint of the scale), χ^2^(1) = 3.92, *p* = .048.

#### Main analyses

Replicating the results of Studies 2 and 3, we again found a positive correlation between faith in intuition and belief in physiognomy, *r*(386) = 0.405, *p* < .001, 95% CI [0.316, 0.485]. The positive correlation still emerged when excluding two items that directly refer to the accuracy of appearance-based impressions from the faith in intuition scale, *r*(386) = 0.326, *p* < .001, 95% CI [0.234, 0.412].

We also found a positive correlation between faith in intuition and participants’ confidence in the accuracy of their trustworthiness impressions based on facial photographs, *r*(386) = 0.260, *p* < .001, 95% CI [0.164, 0.350]. Again, this correlation remained significant when excluding the two items for the faith in intuition scale, *r*(386) = 0.242, *p* < .001, 95% CI [0.146, 0.334].

#### Additional analyses

There was also a positive correlation between belief in physiognomy and participants’ confidence in the accuracy of their trustworthiness impressions, *r*(386) = 0.287, *p* < .001, 95% CI [0.193, 0.376]. Finally, we explored whether individual differences in thinking style and belief in physiognomy are related to two other first impression metrics: response times and judgment extremity. Faith in intuition was not significantly correlated with participants’ average (log-transformed) response time when making the trustworthiness judgments, *r*(386) =  − .025, *p* = .623, 95% CI [− 0.124, 0.075], or with the average extremity of judgments, *r*(386) = 0.063, *p* = .214, 95% CI [− 0.037, 0.162]. There was also no significant association between belief in physiognomy and response times, *r*(386) = 0.024, *p* = .632, 95% CI [− 0.075, 0.124], but we did find a small positive association with the extremity of judgments, *r*(386) = 0.128, *p* = .012, 95% CI [0.029, 0.225].

### Discussion

As in our studies with British and Nigerian participants, the present study with a sample of Dutch university students showed that participants with a more intuitive thinking style more strongly endorsed physiognomic beliefs. Going beyond our previous studies, we also showed that these differences are related to how confident people are in the accuracy of specific character judgments based on a person’s facial appearance. We found that participants with an intuitive thinking style were more confident that their trustworthiness judgments of others based on a facial photograph are accurate.

## General Discussion

In spite of their low accuracy (e.g., Bonnefon et al., 2017; Jaeger et al., 2024), people are confident in their appearance-based first impressions and rely on them when making various decisions (Hassin and Trope, 2000; Olivola et al., 2014). Recent work has started to examine whether this can be explained by lay beliefs in physiognomy (i.e., the idea that a person’s character is reflected in their facial appearance; Madan et al., 2022; Suzuki et al., 2017). Here, we examined the prevalence of physiognomic beliefs and its socio-demographic and psychological correlates. Across four studies, with participants from the United Kingdom, the Netherlands, and Nigeria, we found that belief in physiognomy was relatively common. In each sample, around half of participants at least somewhat endorsed the belief that a person’s character is reflected in their facial appearance. In our large, representative sample of the Dutch population (Study 1), we found that gender, age, education, and income explained little variance in physiognomic beliefs. That is, lay beliefs in physiognomy were similarly prevalent across different demographic groups.

Similar to previous work (Suzuki et al., 2017), we found that physiognomic beliefs were related to the endorsement of more general beliefs about the nature of a person’s character (Studies 1 and 2). Participants who believed that character traits are biologically determined and people who believed in a just world (where dishonest behavior may be associated with a dishonest appearance) were also more likely to endorse physiognomic beliefs. In contrast to previous work (e.g., Madan et al., 2022), we did not find that physiognomic beliefs were positively related to entity beliefs about personality (i.e., the belief that that character traits are a fixed and essential aspect of a person’s identity). Especially Study 1 (with *n* > 2500) was sufficiently powered to detect even very small associations, making it unlikely that we did not obtain previously observed associations due to low power.

It is possible that our results diverged from those of Madan and colleagues (2022), who found a positive correlation for entity beliefs, because of differences in how belief in physiognomy was measured. Whereas our items asked participants about their perceived ability to judge character from facial appearance (“I can learn something about a person’s personality just from looking at his or her face”), Madan and colleagues’ items referenced the existence of a link between a person’ appearance and their character (“You can tell a person’s character from their appearance”). On the one hand, these two sets of items would lead to different scores for people who think that, although they cannot tell a person’s character from their facial appearance, an accurate judgment could be achieved in theory (e.g., by an expert or an algorithm). On the other hand, people’s rich experience with their own first impressions is probably the most salient information that they would draw on when answering both kinds of questions, leading to similar responses on both scales. We would therefore expect the two measures to be strongly correlated, but this remains an open question for future studies. Future studies could also explore other lay beliefs in this domain (e.g., whether people think that some individuals show exceptional face-reading abilities, or that some individuals are easier to read than others, such as individuals from their own culture).

There are also other reasons to doubt that our null results for entity beliefs about personality can be explained by differences between the scales. Suzuki and colleagues (2017) measured belief in physiognomy with items similar to ours (e.g., “I know an immoral person when I see their face”) and found a positive association with entity beliefs with a sample of participants from the United States (similar to Madan et al., 2022). However, this association was smaller and non-significant in two other samples of Japanese and U.S. participants (even though the same number of U.S. participants was recruited via the same company). It appears that the association between physiognomic beliefs and entity beliefs is weaker and less generalizable than other associations reported in the literature.

The main goal of the present studies was to test whether thinking styles explain individual differences in physiognomic beliefs. Trait impressions are formed spontaneously, quickly, and effortlessly (Stewart et al., 2012; Willis and Todorov, 2006), which means that they should be intuitively accessible. Crucially, people vary in how much they generally trust and use their intuitions in judgment and decision-making. We examined the role of different measures of thinking styles and found that physiognomic beliefs were positively related to individual differences in faith in intuition (Epstein et al., 1996), but not to measures of cognitive reflection (Frederick, 2005; Pennycook et al., 2015) or the need to evaluate (Jarvis and Petty, 1996). These results provide suggestive evidence of which cognitive processes may give rise to the endorsement of physiognomic beliefs. It is possible that people who believe in physiognomy do so because it is an intuitively accessible idea that they endorse like other ideas that come to mind intuitively (captured by individual differences in faith in intuition), and not necessarily because it is an automatically held idea that people would reject upon careful reflection (captured by individual differences in cognitive reflection) or because it is a belief that allows people to satisfy their motivation to form quick judgments of others (captured by individual differences in the need to evaluate). The positive association between physiognomic beliefs and faith in intuition still emerged when omitting two items from the faith in intuition scale that directly reference one’s confidence in character judgments, and we found similar results across three studies with British (Study 2), Nigerian (Study 3), and Dutch participants (Study 3).

Previous work has examined correlations between lay beliefs in physiognomy and a host of other lay beliefs and individual differences to understand who holds physiognomic beliefs. However, many of these individual differences may be correlated. For example, we found that participants who put more trust in their intuitions were also more likely to endorse that character traits are biologically determined. For this reason, we also tested which measures still explain variation in the endorsement of physiognomic beliefs when controlling for other measures. When we regressed belief in physiognomy on different lay beliefs and measures of thinking styles, only faith in intuition emerged as a significant predictor. Furthermore, Study 4 showed that faith in intuition was positively associated with participants’ confidence in the accuracy of specific appearance-based character judgments. Together, our findings suggest that individual differences in how much people trust their intuitions are especially important for understanding lay beliefs in physiognomy and confidence in the accuracy of appearance-based impressions.

Our findings converge with previous work on lay personality theory. People hold beliefs about the basis (Haslam et al., 2004), malleability (Chiu et al., 1997), structure (Stolier et al., 2018), and expression (Mehl et al., 2006) of personality traits. These beliefs predict various outcomes related to impression formation (Haslam et al., 2004), information search (Plaks et al., 2001), and stereotyping (Levy et al., 1998). We add to this work by showing that people also hold beliefs about the manifestation of personality traits in facial appearance. Although social perception is often described as a reflexive, stimulus-driven process in which the presence of certain facial cues automatically trigger personality inferences (Engell et al., 2007), recent studies have highlighted that there are many top-down processes that influence social perception (Brambilla et al., 2018; Stolier et al., 2018). Our findings are in line with this view and underline the important role of conceptual beliefs in impression formation. Specifically, Study 4 showed that individual differences in physiognomic beliefs were related to the confidence and extremity of people’s impressions.

## Limitations and Future Directions

Although we replicated our main finding, the positive association between faith in intuition and lay beliefs in physiognomy, across three studies (two preregistered) with participants from the United Kingdom, the Netherlands, and Nigeria, the robustness and generalizability of this and other findings warrant further testing. Based on the present results, we suggest that even though many lay beliefs and trait measures may be correlated with physiognomic beliefs, our studies indicate that faith in intuition is particularly relevant for explaining variation in physiognomic beliefs. This inference is based on the observation that among all the measures we examined, faith in intuition showed the strongest correlation and was the only construct that showed a significant association when all variables were entered as predictors of physiognomic beliefs in the same regression model. However, it is possible that other individual differences that we did not consider here ultimately show stronger unique associations with physiognomic beliefs (e.g., belief in free will; Madan et al., 2022).

We found that physiognomic beliefs were similarly prevalent across different sociodemographic groups in a large sample of the Dutch general population. Whereas previous investigations showed that lay beliefs in physiognomy were relatively common among participants from the United States and Japan (Madan et al., 2022; Suzuki et al., 2017), we found similar results with British, Nigerian, and Dutch participants. Our results provide further evidence that lay beliefs in physiognomy are widespread in many countries. However, additional work is needed to more systematically explore potential differences in the prevalence of physiognomic beliefs across cultures. Future studies could, for example, examine whether similar patterns in the prevalence, structure, and correlates of physiognomic belief can be observed in small-scale societies, or whether there are cultural factors that can explain cross-country differences.

The focus of the present studies was to explore which trait measures best explain endorsement of physiognomic beliefs. An open question is whether the links with trait measures observed here are also mirrored by similar links with state measures. That is, given the observed association between faith in intuition and belief in physiognomy, it is plausible that manipulations which increase or decrease how much people think they can trust their intuitions would also affect belief in physiognomy. Testing this prediction might be difficult. It is questionable whether beliefs that have been held and reinforced for many years, such as the belief that one’s intuitions are usually accurate, can be changed in a typical lab experiment to such an extent that resulting changes in physiognomic belief are detectable with common sample sizes. Even though manipulations of some beliefs (e.g., growth versus. fixed mindset) with brief interventions (e.g., reading a short scientific article that presents evidence for or against the belief) are common in the literature, recent work questions their validity (Macnamara and Burgoyne, 2023; Sisk et al., 2018). Many studies that manipulated growth mindset did not test whether the manipulation changed participants’ beliefs and in the studies that included manipulation checks, only about half found a significant effect of the manipulation (Sisk et al., 2018). Research also suggests that it is difficult to reduce people’s reliance on their intuitions and first impressions (Jaeger et al., 2020; Roy et al., 2025; Simmons and Nelson, 2006). These examples demonstrate that changing fundamental beliefs (to a large enough degree that resulting changes in some outcome variable can be detected with realistic sample sizes), such as the belief that intuitions provide mostly valid insights, is probably more difficult than previously assumed and manipulations should be carefully tested and validated.

Similarly, future studies could also test whether the present insights can be used to reduce people’s unwarranted confidence in the accuracy of their appearance-based trait impressions (Hassin and Trope, 2000; Jaeger et al., 2024). Educating people about the fact that information that is intuitively accessible is not necessarily accurate (West, 2010) may help people realize that their first impressions are often less accurate that they think. Similar effects may be achieved by giving people repeated feedback on their accuracy in a first impression test to demonstrate that they may be overconfident (Israelashvili and Karniol, 2019). More evidence is needed to test whether such interventions have a longer-lasting effect on lay beliefs in physiognomy and whether they also reduce how much people rely on trait impressions in judgments and decision-making.

## Conclusion

The present studies tested who believes in physiognomy, the idea that a person’s character is reflected in their facial appearance. Our findings highlight that such beliefs are common (in all samples, around half of participants at least somewhat endorsed it) and similarly prevalent across different socio-demographic groups (i.e., gender, age, education, and income). Replicating previous studies, we found that physiognomic beliefs were positively related to other lay beliefs about the nature of personality traits (e.g., belief in their biologically determined nature). Extending previous studies, we found that physiognomic beliefs were most strongly and consistently related to how much people trust their intuitions, and this finding replicated in samples of British, Nigerian, and Dutch participants. We also found that participants who scored higher on faith in intuition were more confident in the accuracy of their appearance-based trustworthiness impressions. These findings are in line with the idea that the diagnostic value of first impressions is intuitively appealing because of the quick and effortless way in which they emerge (Stewart et al., 2012; Willis and Todorov, 2006), Taken together, the present studies suggest that individual differences in the endorsement physiognomic beliefs (and how confident people are in the accuracy of their impressions) can be explained by variation in how much people generally trust their intuitions.

## Supplementary Information

Below is the link to the electronic supplementary material.

Supplementary Material 1

## Data Availability

All data, analysis scripts, and preregistration documents are available at the Open Science Framework (https://osf.io/s9nj8/).

## Footnotes

1 The evidence for a positive association with entity beliefs about personality is more mixed. Madan and colleagues (2022) found a positive association (*r* = .0.29) with U.S. participants when testing if entity beliefs moderate the relation between belief in physiognomy and support for facial profiling technology. Suzuki and colleagues (2017) found a positive associations for U.S. participants in one of their studies (*r* = .0.19), but the association was not significant for Japanese participants (*r* = .0.01) or in another study with U.S. participants (*r* = .0.09).

2 In all four studies, we also measured attribute-specific physiognomic beliefs for exploratory purposes. We asked participants how accurately they could judge a person on various characteristics from looking at that person’s passport photo. For each item, responses were indicated using a slider from 0 (not at all accurately) to 100 (extremely accurately). Participants responded to twelve items: Three sociability-related traits (*warmth, friendliness, likeability*), three morality-related traits (*trustworthiness, sincerity, honesty*), and three competence-related traits (*competent*, *intelligent*, *skillful*; Brambilla et al., 2011). We also included three additional, physically salient characteristics—*gender*, *age*, and *attractiveness*. The order in which the twelve items were presented was randomized. Results for this measure are presented in the Supplemental Materials.

3 When analyzing trait-specific physiognomic beliefs, correlations ranged from *r* = 0.426 for age-specific belief to *r* = 0.721 for competence-specific belief. Meta-analyzing the twelve trait-specific correlations yielded an average of *r* = 0.609.
