# Supplementary material for: Intuitive Thinking is Associated with Stronger Belief in Physiognomy and Confidence in the Accuracy of Facial Impressions
Source: J Nonverbal Behav. 2025 Oct 4;49(4):505–27. doi: 10.1007/s10919-025-00497-w (PMC12627204; doi:10.1007/s10919-025-00497-w)
Supplement: Supplementary file 1 — Supplementary Material 1 [file 10919_2025_497_MOESM1_ESM.docx]

# Supplemental Materials

Here, we report additional results that were omitted from the main manuscript for the sake of brevity.

# Study 1: Additional Analyses

In Study 1, we examined relations between physiognomic belief and various demographic characteristics (i.e., sex, age, income, and level of education). We regressed general physiognomic belief on gender (coded 0 for male and 1 for female), age, income (*z*-standardized net monthly income), and level of education (six levels, ranging from primary school to university degree; see Table S1, Model 1). Women scored higher on physiognomic belief than men, *β* = 0.104, *SE* = 0.048, *t*(2,335) = 2.17, *p* = .030, 95% CI [0.010, 0.198], there was a negative effect of age, *β* = -0.004, *SE* = 0.001, *t*(2,335) = 3.07, *p* < .001, 95% CI [-0.007, -0.002], but no significant effect of income, *β* = 0.166, *SE* = 0.153, *t*(2,335) = 1.08, *p* = .28, 95% CI [-0.135, 0.466], We also found no significant differences in physiognomic belief across different education levels. Compared to a primary school education, physiognomic belief was not significantly different for participants who had finished a preparatory secondary vocational education, *β* = 0.055, *SE* = 0.105, *t*(2,335) = 0.53, *p* = .60, 95% CI [-0.151, 0.262], a senior general secondary education, *β* = -0.055, *SE* = 0.119, *t*(2,335) = 0.46, *p* = .65, 95% CI [-0.289, 0.179], a secondary vocational education, *β* = 0.053, *SE* = 0.105, *t*(2,335) = 0.50, *p* = .61, 95% CI [-0.153, 0.259], a university of applied sciences education, *β* = 0.042, *SE* = 0.107, *t*(2,335) = 0.40, *p* = .69, 95% CI [-0.166, 0.251], or an academic university education, *β* = -0.154, *SE* = 0.118, *t*(2,335) = 1.30, *p* = .19, 95% CI [-0.387, 0.078]. In Model 2 (Table S1), we added belief in biological determinism and entity beliefs as additional predictors.

## Studies 1-4: Additional Analyses of Physiognomic Beliefs

First, we tested whether people believe that personality traits are less visible in faces than characteristics that are less perceptually ambiguous (i.e., gender, age, and attractiveness). In Study 1, personality-specific physiognomic beliefs (*M* = 32.12, *SD* = 18.70) were significantly weaker than physiognomic beliefs for gender (*M* = 75.66, *SD* = 19.48), *t*(2,623) = 83.45, *p* < .001, *d* = 2.28, 95% CI [2.18, 2.38], age (*M* = 60.52, *SD* = 17.41), *t*(2,623) = 62.52, *p* < .001, *d* = 1.57, 95% CI [1.50, 1.65], and attractiveness (*M* = 64.59, *SD* = 20.68), *t*(2,623) = 65.59, *p* < .001, *d* = 1.65, 95% CI [1.57, 1.72].

In Study 2, personality-specific physiognomic beliefs (*M* = 21.75, *SD* = 19.14) were significantly weaker than physiognomic beliefs for gender (*M* = 81.79, *SD* = 21.66), *t*(228) = 37.05, *p* < .001, *d* = 2.92, 95% CI [2.57, 3.28], age (*M* = 69.30, *SD* = 17.13), *t*(228) = 33.11, *p* < .001, *d* = 2.61, 95% CI [2.28, 2.93], and attractiveness (*M* = 73.51, *SD* = 22.96), *t*(228) = 32.46, *p* < .001, *d* = 2.43, 95% CI [2.14, 2.72].

In Study 3, personality-specific physiognomic beliefs (*M* = 34.17, *SD* = 18.33) were significantly weaker than physiognomic beliefs for gender (*M* = 78.58, *SD* = 21.56), *t*(387) = 35.06, *p* < .001, *d* = 2.21, 95% CI [1.98, 2.44], age (*M* = 74.02, *SD* = 16.06), *t*(387) = 35.01, *p* < .001, *d* = 2.31, 95% CI [2.06, 2.55], and attractiveness (*M* = 78.90, *SD* = 19.09), *t*(387) = 39.43, *p* < .001, *d* = 2.38, 95% CI [2.15, 2.62].

Finally, in Study 4, personality-specific physiognomic beliefs (*M* = 36.08, *SD* = 22.31) were significantly weaker than physiognomic beliefs for gender (*M* = 88.42, *SD* = 20.44), *t*(146) = 21.79, *p* < .001, *d* = 2.43, 95% CI [2.00, 2.87], age (*M* = 65.40, *SD* = 25.46), *t*(146) = 13.18, *p* < .001, *d* = 1.22, 95% CI [0.98, 1.45], and attractiveness (*M* = 78.07, *SD* = 22.31), *t*(146) = 17.04, *p* < .001, *d* = 1.83, 95% CI [1.48, 2.17].

We also compared the endorsement of physiognomic beliefs for different personality dimensions. In Study 1, we found that participants held stronger physiognomic beliefs for sociability (*M* = 38.42, *SD* = 21.36) than for morality (*M* = 29.65, *SD* = 20.49), *t*(2,623) = 34.56, *p* < .001, *d* = 0.42, 95% CI [0.39, 0.44], and competence (*M* = 28.29, *SD* = 18.76), *t*(2,623) = 34.80, *p* < .001, *d* = 0.59, 95% CI [0.47, 0.53], . Morality-specific physiognomic beliefs were stronger than competence-specific physiognomic beliefs, but the difference was smaller, *t*(2,623) = 5.77, *p* < .001, *d* = 0.07, 95% CI [0.05, 0.09].

In Study 2, we found that participants held stronger physiognomic beliefs for sociability (*M* = 29.85, *SD* = 23.84) than for morality (*M* = 19.30, *SD* = 20.18), *t*(228) = 11.98, *p* < .001, *d* = 0.46, 95% CI [0.38, 0.54], and competence (*M* = 16.11, *SD* = 16.97), *t*(228) = 14.42, *p* < .001, *d* = 0.60, 95% CI [0.51, 0.69]. Morality-specific physiognomic beliefs were stronger than competence-specific physiognomic beliefs, but the difference was smaller, *t*(228) = 4.62, *p* < .001, *d* = 0.16, 95% CI [0.09, 0.23].

In Study 3, we found that participants held stronger physiognomic beliefs for sociability (*M* = 47.77, *SD* = 21.94) than for morality (*M* = 30.37, *SD* = 20.94), *t*(387) = 20.38, *p* < .001, *d* = 0.81, 95% CI [0.72, 0.90], and competence (*M* = 24.38, *SD* = 19.08), *t*(387) = 25.10, *p* < .001, *d* = 1.13, 95% CI [1.01, 1.24]. The difference between morality-specific beliefs and competence-specific beliefs was not significant, *t*(146) = 0.15, *p* < .001, *d* = 0.01, 95% CI [-0.08, 0.10].

Finally, in Study 4, we found that participants held stronger physiognomic beliefs for sociability (*M* = 29.85, *SD* = 23.84) than for morality (*M* = 19.30, *SD* = 20.18), *t*(228) = 11.98, *p* < .001, *d* = 0.46, 95% CI [0.38, 0.54], and competence (*M* = 16.11, *SD* = 16.97), *t*(228) = 14.42, *p* < .001, *d* = 0.60, 95% CI [0.51, 0.69]. Morality-specific physiognomic beliefs were stronger than competence-specific physiognomic beliefs, but the difference was smaller, *t*(384) = 8.14, *p* < .001, *d* = 0.30, 95% CI [0.22, 0.37]. Thus, replicating earlier work (Jaeger et al., 2022), we found that people believe that a person’s sociability is more reflected in their facial appearance than their morality or competence. Thus, across all four studies and replicating earlier work (Jaeger et al., 2022), we found that people believe that a person’s sociability is more reflected in their facial appearance than their morality or competence.

**References**

Jaeger, B., Evans, A. M., Stel, M., & van Beest, I. (2022). Understanding the role of faces in person perception: Increased reliance on facial appearance when judging sociability. *Journal of Experimental Social Psychology*, *100*, 104288. https://doi.org/10.1016/j.jesp.2022.104288
